# Testing of UK Populations of *Culex pipiens* L. for Schmallenberg Virus Vector Competence and Their Colonization

**DOI:** 10.1371/journal.pone.0134453

**Published:** 2015-08-20

**Authors:** Robyn Manley, Lara E. Harrup, Eva Veronesi, Francesca Stubbins, Jo Stoner, Simon Gubbins, Anthony Wilson, Carrie Batten, Constantianus J. M. Koenraadt, Mark Henstock, James Barber, Simon Carpenter

**Affiliations:** 1 Vector-borne Viral Diseases Programme, The Pirbright Institute, Pirbright, Surrey, United Kingdom; 2 Laboratory of Entomology, Wageningen University and Research (WUR), Wageningen, The Netherlands; University of California Davis, UNITED STATES

## Abstract

**Background:**

*Schmallenberg virus* (SBV), an arboviral pathogen of ruminants, emerged in northern Europe during 2011 and has subsequently spread across a vast geographic area. While *Culicoides* biting midges (Diptera: Ceratopogonidae) have been identified as a biological transmission agent of SBV, the role of mosquitoes (Diptera: Culicidae) as potential vectors has not been defined beyond small-scale field collections in affected areas. *Culex pipiens* L. are one of the most widespread mosquitoes in northern Europe; they are present on farms across the region and have previously been implicated as vectors of several other arboviruses. We assessed the ability of three colony lines of *Cx*. *pipiens*, originating from geographically diverse field populations, to become fully infected by SBV using semi-quantitative real-time RT-PCR (sqPCR).

**Findings:**

Two colony lines of *Cx*. *pipiens* were created in the UK (‘Brookwood’ and ‘Caldbeck’) from field collections of larvae and pupae and characterised using genetic markers. A third strain of *Cx*. *pipiens* from CVI Wageningen, The Netherlands, was also screened during experiments. Intrathoracic inoculation of the Brookwood line resulted in infections after 14 days that were characterised by high levels of RNA throughout individuals, but which demonstrated indirect evidence of salivary gland barriers. Feeding of 322 individuals across the three colony lines on a membrane based infection system resulted in no evidence of full dissemination of SBV, although infections did occur in a small proportion of *Cx*. *pipiens* from each line.

**Conclusions/Significance:**

This study established two novel lines of *Cx*. *pipiens* mosquitoes of UK origin in the laboratory and subsequently tested their competence for SBV. *Schmallenberg virus* replication and dissemination was restricted, demonstrating that *Cx*. *pipiens* is unlikely to be an epidemiologically important vector of the virus in northern Europe.

## Introduction

In 2011 a novel *Orthobunyavirus* of ruminants, provisionally named *Schmallenberg virus* (SBV) was identified through metagenomic analysis in Germany [1]. *Culicoides* biting midges (Diptera, Ceratopogonidae) were rapidly identified by several groups as being the primary biological vector of SBV through field studies [2,3]. These investigations were aided by the standardization of diagnostic tools for detection of SBV RNA in putative vector *Culicoides* species using colony lines [4]. Since this identification, the vector competence of alternative groups, including mosquitoes and ticks, has not been assessed in detail in the laboratory using colony derived lines, although several studies have failed to find SBV in field collections of mosquitoes [5,6]. This is despite the demonstration that at least one other member of the *Orthobunyavirus* genus, Oropouche virus, possesses the ability to infect and replicate to full dissemination in both *Culicoides* and mosquitoes under laboratory conditions [7,8].

In Europe, *Culex pipiens* L. is the most common and widespread mosquito inhabitant of small water bodies (including containers and ponds within urban areas) [9], although conflation with the morphologically similar *Cx*. *torrentium* Martini remains an issue in mapping distribution [10]. The phylogenetics of the Pipiens complex (*Cx*. *pipiens*, *Cx*. *quinquefasciatus* Say, *Cx*. *australicus* Dobrotworsky and Drummond, *Cx*. *globocoxitus* (Dobrotworsky and Drummond) and *Cx*. *pallens* (Coquillett)), has been the subject of investigation worldwide due to their role in the biological transmission of arboviruses [11,12]. There is still continued debate, however, regarding the specific status and relationship between the members of this complex [12]. In Europe, *Cx*. *pipiens* is the sole representative of the Pipiens complex and is observed in two ecological forms; *Cx*. *pipiens* form pipiens and *Cx*. *pipiens* form molestus. These ecological forms are morphologically inseparable and also found in other regions of the world [11]. Recently, hybrids between these two forms in Europe and North Africa have also been identified [13,14] and these have been shown to be present and sympatric with both forms recorded in the United Kingdom [15]. It has been suggested that hybridization in *Cx*. *pipiens* may, alongside differences in host preference [16], drive transmission of West Nile virus (WNV; *Flaviviridae*, *Flavivirus*) through hybrids possessing a greater vector competence [17]. The full characterization of individuals as specific forms or hybrids is therefore becoming an increasing important factor in studies [15].

In this paper we provide an account of the colonization and subsequent molecular characterization of two strains of *Culex pipiens* L. derived from field material collected in the UK. We then investigate vector competence for SBV using these two colony lines and an additional line from The Netherlands, through a combination of intrathoracic inoculation and oral feeding of blood/SBV suspensions through a membrane-based system. In doing so we provide baseline data for further studies of SBV in field-collected mosquitoes and a direct comparison with levels of infection in *Culicoides* under laboratory conditions, which have been conducted in previous laboratory studies [4].

## Methods

### Colonization of mosquitoes

Two colony lines of *Cx*. *pipiens* designated ‘Caldbeck’ and ‘Brookwood’ were established from field populations located in the UK during May 2011. Several hundred mosquito larvae and pupae were collected from a suburban freshwater pond in Surrey, United Kingdom (51.3809N, -0.2390W) to initiate the Caldbeck line. A similar number of mosquito larvae and pupae were also collected from a container habitat in an allotment area in Surrey (51.3065N, -0.6328W). Mosquito larvae and pupae collected from the field sites were transferred in water from their original habitat to The Pirbright Institute and colonization was conducted at 25 ±1°C with a photo period of 16:8 (light: dark) hours in a contained insectary room. Mosquitoes were allowed to emerge from the container and mate freely in a 32.5 x 32.5 x32.5 cm white net cage (MegaView Science Co. Ltd, Taichung, Taiwan).

Blood feeding of mosquitoes of mixed age was then conducted overnight (exposure from approximately 16.00–09.00h) using a Hemotek system (Hemotek Ltd, UK), with a stretched Parafilm M (Bermis Company, INC., USA) membrane and defibrinated horse blood (TCS Biosciences, UK) heated to 37°C. One black plastic cup (425 ml capacity) containing 125 ml of tap water, which had been allowed to stand for at least 24 hours to allow any chlorine to evaporate, was provided for oviposition from day four post-feeding and egg rafts were collected for up to two weeks after the initial blood-meal. This cycle of blood-feeding and egg collection was repeated on average six times per generation (range 5–12).

Egg rafts and first instar larvae were transferred to a larval development bowl which contained approximately 1.5L of water and ¼ teaspoon of ground Mr Johnson’s Advance Guinea Pig Food (Mr Johnson’s, UK) added each Monday, Wednesday and Friday. Ten to twelve egg rafts were introduced into each bowl (resulting in approximately 500–800 emerging 1^st^ instar larvae) and the rearing bowl was covered with netting to prevent the release of emerging adults. Larvae required approximately 10–14 days to develop, pupate and emerge as adults and both pupae and adults were collected and placed in clean cages at a density of up to 500 individuals/cage. Sucrose solution (10% w/v) was provided to adults via a cotton wool pad placed in a 6cm diameter sample pot lid.

An additional established line of *Cx*. *pipiens* complex mosquitoes was provided from The Netherlands, designated ‘Wageningen’. This line was created in August 2010 from egg rafts collected from a metal water-holding tub in a domestic garden. The colony was maintained on fresh chicken blood using Hemotek blood feeders as described above. Prior to shipping the colony line was maintained at 23 ±1°C and a photoperiod of 16:8 light: dark. Egg rafts were shipped to Pirbright in March 2012 and then maintained under the conditions described for colonies maintained at Pirbright.

### Molecular characterization of mosquitoes

Mosquitoes were initially characterized using morphology of emerging adults which restricted candidate species to *Cx*. *pipiens* and *Cx*. *torrentium*. A series of molecular assays were then used to characterize colony lines to species and ecological form in the original field material (n = 5 ♀ for both lines), first generation (n = 5 ♀ for both lines) and the 19^th^ (Caldbeck) or 20^th^ generation (Brookwood) (n = 40 (20 ♂, 20♀) for both lines). In the individuals from the 19^th^ (Caldbeck) and 20^th^ (Brookwood) generations, extraction of DNA was carried out using three legs from each specimen and the remaining bodies were subsequently prepared as pinned morphological voucher specimens. In field and first generation specimens that had been stored at -20°C in 70% ethanol, DNA was extracted from decapitated whole mosquitoes.

DNA extraction was carried out using specimens placed in 1.5ml microcentrifuge tubes with 180μl PBS and homogenized using a disposable pellet pestle (Sigma-Aldrich, Gillingham, UK) for 30 seconds. Twenty μl of proteinase K and 180μl Qiagen Buffer AL (Qiagen, Crawley, UK) were added to the resulting homogenate and incubated at 56°C for 10 minutes using a dry block heater (Techne Dri-Block DB-2P: Bibby Scientific Ltd, Stone, UK), 200μl of 100% ethanol was then added to each sample and mixed thoroughly by vortexing (Genie 2 Vortex: Scientific Industries Inc., Bohemia, NY, USA). Total DNA was extracted and purified from the resulting homogenate using a DNeasy Blood & Tissue Kit (Qiagen).

Assays used for characterization of mosquitoes are described in Table 1 and conducted in the order given. All polymerase chain reactions (PCR’s) were conducted using a GeneAmp 9700 thermal cycler (Applied Biosystems, Foster City, CA, USA) and negative controls for the amplification reactions were carried out at every PCR round. Reactions were stored at 4°C prior to visualization. Amplification success for the COI and *ace*-2 assays was assessed using 2% (w/v) E-Gel 96 gels containing SYBR Safe (Invitrogen, Paisley, UK) run for 8 minutes on program EP. Results of the CQ11 microsatellite assay were visualized using 2% agarose gels containing SYBR Safe DNA Gel Stain (Invitrogen, Paisley, UK) run for 30 minutes at 100 volts. Bands were identified by comparison with E-Gel Low Range Quantitative DNA Ladder (100–2000bp: Invitrogen). For lanes containing the duplex *ace*-2 PCR products fragment sizes identified *Cx*. *pipiens* (610bp fragment) or *Cx*. *torrentium* (416bp fragment). For lanes containing the duplex CQ11 microsatellite locus PCR products fragment sizes were identified as *Cx*. *pipiens* f. pipiens (180bp fragment), Cx. *pipiens* f. molestus (250bp fragment) or *Cx*. *pipiens* f. pipiens x f. molestus (presence of both 180bp and 250bp fragments).

**Table 1:** Polymerase chain reaction (PCR)-based assays used for genetic characterization of Brookwood and Caldbeck *Culex pipiens* lines.

Assay	PCR Reaction Mix	PCR Conditions
**COI Barcode assay [41]**	Reaction volume of 25μl: 2.5μl nuclease-free water (Qiagen, UK); 12.5μl Qiagen TopTaq Master Mix (Qiagen, UK); 2.5μl CoralLoad Concentrate (Qiagen, UK); 1.25μl of the 10μM forward primer LCO1490 (5’-GGTCAACAAATCATAAAGATATTGG-3’); 1.25μl of the 10μM reverse primer HCO2198 (5’-TAAACTTCAGGGTGACCAAAAAATCA-3’); 5.0μl of template DNA per reaction.	Denaturation at 94°C for three minutes; 35 cycles 94°C for 30 seconds; 46°C for 30 seconds; 72°C for one minute. Final extension step 72°C for ten minutes.
**Eurasia *ace*-2 multiplex assay [42]**	Reaction volume of 10μl: 0.4μl nuclease-free water (Qiagen, UK); 5.0μl Qiagen TopTaq Master Mix (Qiagen, UK); 0.2μl 25mM Magnesium Chloride (MgCL_2_) (Qiagen, UK); 1.0μl CoralLoad Concentrate (Qiagen, UK); 0.1μl of 10μM forward primer ACEtorr (5’- TGCCTGTGCTACCAGTGATGTT -3’; 0.1μl of 10μM forward primer ACEpip (5’- GGAAACAACGACGTATGTACT -3’; 0.2μl of 10μM reverse primer B1246s (5’-TGGAGCCTCCTCTTCACGG-3’; 3.0μl of template DNA per reaction.	Denaturation at 94°C for three minutes; 35 cycles of 94°C for 30 seconds, 55°C for 30 seconds, 72°C for one minute. Final extension step 72°C for ten minutes.
**CQ11 microsatellite assay [22]**	Reaction volume of 20μl: 0.80μl nuclease-free water (Qiagen, UK); 10.0μl Qiagen TopTaq Master Mix (Qiagen, UK); 0.4μl 25mM Magnesium Chloride (MgCL_2_) (Qiagen, UK); 0.15μl 20mg/ml Bovine Serum Albumin (Sigma-Aldrich Company Ltd, UK); 2.0μl CoralLoad Concentrate (Qiagen, UK); 0.15μl of 10μM forward primer CQ11F (5’- GATCCTAGCAAGCGAGAAC-3’; 0.20μl of 10μM reverse primer pipCQ11R (5’- CATGTTGAGCTTCGGTGAA -3’; 0.30μl of 10μM reverse primer molCQ11R (5’-CCCTCCAGTAAGGTATCAAC-3’; 6.0μl of template DNA per reaction.	Denaturation 95°C for three minutes; 40 cycles of 94°C for 30 seconds, 55°C for 30 seconds, 72°C for one minute. Final extension step at 72°C for ten minutes.

Sequencing of the COI Barcode region [18] was performed on PCR product purified using a MinElute PCR purification kit (Qiagen). DNA concentration following purification was estimated using a NanoDrop 2000 UV-Vis Spectrophotometer (Fisher Scientific UK, UK), then diluted using 10 mM Tris-HCL pH 8.0 (Buffer EB: Qiagen) to 1ng per μl per 100 bases (~7ng/μl) and sent for bi-directional sequencing at a commercial facility (Source BioScience, UK) using primers HCO2198 and LCO1490 (3.2μM). Sequences were edited and assembled using CodonCode Aligner (CodonCode Aligner, Centerville, MA, USA) and consensus sequences aligned using MUSCLE [19]. These sequences were compared to previously published sequences using BLAST in MEGA version 5.2 [20].

Morphological specimens were vouchered by pinning using the double mounted method [21]. Prior to pinning, the last three segments of the male abdomens were removed using a microscapel and cleared by incubating individually in 200μl of 10% w/v KOH for 20 minutes at 6°C using a dry block heater (Techne Dri-Block DB-2P). Following incubation phallosomes were removed and transferred to acetic acid for 5 minutes to neutralize any remaining KOH. The phallosomes were then dehydrated in 100% ethanol for 20 minutes and transferred to soften in 1:1 clove oil: 100% ethanol mix for 20 minutes and transferred to 100% clove oil for at least 20 minutes prior to mounting. The genitalia were then slide mounted in Euparal with the ventral surface facing upwards with the dististyles extended. Digital images were taken using a QICAM Fast 1394 digital camera (QImaging, Surrey, BC, Canada) and Image-Pro Insight (MediaCybernetics, Rockville, MD, USA) mounted on a Leica M80 stereo light microscope (Leica Microsystems, Milton Keynes, UK) for pin-mounted specimens and on a Nikon Alphaphot-2 compound light microscope (Nikon UK Ltd, Kingston Upon Thames, Surrey, UK) for slide-mounted specimens.

*Culex pipiens* specimens from the Wageningen line were also characterized using morphological identification to separate them from specimens of *Cx*. *torrentium* [22]. Egg rafts had originally collected from an above-ground location (typical for *Cx*. *pipiens* f. pipiens), but were not morphologically vouchered. Two years following initial colonization, a total of 64 individuals were processed using the CQ11 microsatellite assay to define *Cx*. *pipiens* forms present in the line [22].

### Infection studies with SBV

The SBV strain used for studies of infection of mosquitoes has been previously described in detail and was originally provided by the Friedrich-Loeffler-Institute, Isle of Riems, Germany [4]. All infection studies were carried out using batches of approximately 40–50, 2–3 day old adult *Cx*. *pipiens* from the 11^th^ generation of the two UK colony lines (Brookwood and Caldbeck) and an unknown generation of the Wageningen line.

### Intrathoracic Inoculation

Approximately 200 individuals of the Brookwood line were inoculated intrathoracically (IT) using 0.4μl of SBV suspension and a micro-injector equipped with a foot driver (Drummond Scientific Nanoject II: Drummond Scientific, USA). The SBV strain had been passaged once through a *Culicoides sonorensis* Wirth and Jones cell line and four times through a Baby Hamster Kidney (BHK-21) cell line (C_q_ value 10–12; infectivity on BHK-21 cells of 5–5.5 log_10_TCID_50_). Ten of the IT inoculated Brookwood line were processed immediately by sqPCR using an identical assay to a previous study [4], and the remainder incubated for 14 days at 25 ± 1°C with access to 10% sucrose solution.

Following incubation, 35 Brookwood line mosquitoes were processed as whole insects using sqPCR and a further 8 were used to determine if SBV infected the salivary glands and was secreted through eliciting salivation and dissection. In these individuals, a drop of pillocarpine (parasympathomimetic alkaloid: Sigma Aldrich, UK) solution (1:4 diluted) was applied to the ventral surface of each mosquito and saliva collected into a 1 μl microcapillary glass tube containing 10% fetal bovine serum (FBS). The collected media was then expelled into individual Eppendorf tubes containing 0.5ml of Schneider’s *Drosophila* Media (Gibco: Life Technologies, Paisley, UK) and 10% FBS supplemented with 1000 IU/ml Penicillin/Streptomycin and 4μg/ml Amphotericin B, before processing in duplicate using sqPCR. Each of these eight individuals was then dissected into head and abdomen/thorax and processed using sqPCR. The C_q_ values resulting from testing were then used to infer SBV RNA quantity in samples using means of the two C_q_ values. Where one of the duplicated samples did not give C_q_ value while the other did, the single value was used.

### Membrane-based Infections

*Culex pipiens* mosquitoes from all three lines were then fed on defibrinated sheep-blood (TCS Biosciences, UK) / SBV suspensions via the Hemotek system (Hemotek Ltd, UK) and a Parafilm M membrane (Bermis Company, INC., USA). A 1:1 mixture of blood and virus was used throughout the experiments and resulted in blood meals of C_q_ 14–15. Five membrane fed Brookwood line and three Caldbeck line mosquitoes were processed immediately for SBV RNA and the remainder incubated for 14 days at 25 ±1°C with access to 10% sucrose solution. Following incubation, 322 individuals were processed for SBV RNA as whole insects (114 from the Brookwood line; 85 from the Caldbeck line and 123 from the Wageningen line). The remaining 18 from the Caldbeck line were treated as for the dissemination experiment in the earlier IT inoculation study.

### Processing of samples for sqPCR

Whole *Cx*. *pipiens*, decapitated bodies and heads were homogenized individually for 1 min at 25hz in 100μl of Schneider’s *Drosophila* Media (Gibco: Life Technologies, Paisley, UK) containing 10% FBS using a TissueLyser (Qiagen, UK) and 3mm stainless steel beads (Dejay Distribution Ltd., UK). Nucleic acid extraction of SBV from samples was subsequently carried out using a Universal Biorobot (Qiagen, UK) in a 96-well format using a QIAamp All Nucleic Acid MDx Kit (Qiagen, UK). Schmallenberg virus RNA in *Cx*. *pipiens* samples was quantified using a sqPCR that targeted the S segment of the genome [23]. Comparisons of RNA quantities were made using C_q_ values generated from samples.

### Statistical analyses

Statistical differences in C_q_ values were assessed using comparison of confidence intervals and non-parametric Wilcoxon rank-sum and Kruskal-Wallis tests.

### Ethics statement

All three *Cx*. *pipiens* colony lines were created from material taken from privately owned land related to the authors and further collections should be requested from the corresponding author (SC). Blood used during experiments and in routine colony maintenance was supplied by TCS biosciences for the Brookwood and Caldbeck lines in the UK and chicken blood was taken from the commercial Kemperkip slaughterhouse in The Netherlands. Use of blood from these sources does not require ethical permission or licensing in either the UK or in The Netherlands.

## Results

### Colonization and molecular characterisation of UK *Cx*. *pipiens* lines

Both UK lines of *Cx*. *pipiens* were colonized with what anecdotally appeared to be a relatively limited bottleneck in the F_1_ and F_2_ generations. Results of the molecular assays used to characterize the Brookwood and Caldbeck *Cx*. *pipiens* lines are summarized in Table 2. Indels, frame shifts and stop codons were absent among COI sequences and their translations, suggesting pseudogenes were not present in the alignment. In contrast to a previous study [24], the nucleotide substitution of a G for an A at the 3rd position of the 68^th^ codon of the COI gene fragment in *Cx*. *pipiens* f. molestus when compared to *Cx*. *pipiens* f. pipiens specimens did not occur. Morphological images and DNA sequences including electropherograms have been made publically available via the Barcode of Life Data System (BOLD) [25] under project PIRCX (dx.doi.org/10.5883/DS-PIRCX) and DNA sequences are also available on GenBank (accession numbers Brookwood: F_0_ *-* KM439036-KM439038, KM439054, KM439055, F_1_-KM439041-KM439045 and F_20_-KM438957-KM438995, KM439040; Caldbeck, F_0_ *-* KM439046-KM439050, F_1_- KM439039, KM439051-KM439053 and F_19_-KM438996-KM439035). The exact generation of the Wageningen *Cx*. *pipiens* line was unknown, but the line had been in rearing for > 2 years at the time of analysis.

**Table 2:** Species and form identification of mosquito lines used during infection studies determined using COI sequence identity, and CQ11 microsatellite and *ace*-2 multiplex assays (* carried out through CQ11 assay only on individuals of an unrecorded generation > 2 years following colonization).

Colony line	Generation	n	*Culex torrentium*	*Cx*. *pipiens* form pipiens	*Cx*. *pipiens* form molestus	*Cx*. *pipiens* hybrid
**Brookwood**	**Field**	5	2	2	0	1
	**F**_**1**_	5	0	4	0	1
	**F**_**20**_	40	0	10	5	25
**Caldbeck**	**Field**	5	0	5	0	0
	**F**_**1**_	5	0	5	0	0
	**F**_**19**_	40	0	40	0	0
**Wageningen**	F*	64	-	5	47	12

### Infection studies with SBV

#### Intrathoracic Inoculation

**Fig 1:**
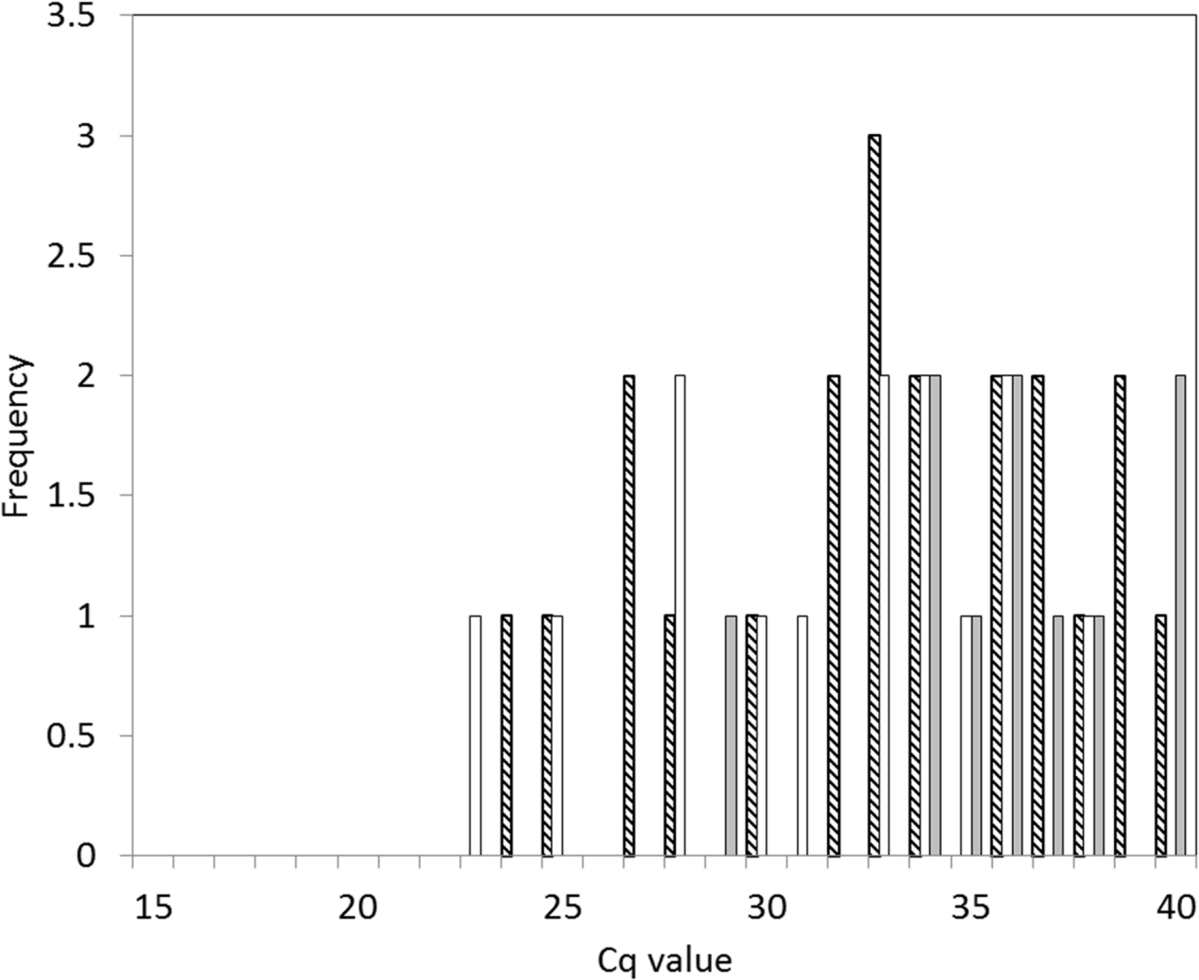
Frequency histogram of responses of *Cx*. *pipiens* lines to attempted infection with Schmallenberg virus. Black = Intrathoracically inoculated Brookwood line; Cross-hatch = Orally fed Brookwood line; White = Orally fed Caldbeck line; Grey = Orally fed Netherlands line. Samples of >40 C_q_ are excluded.

**Fig 2:**
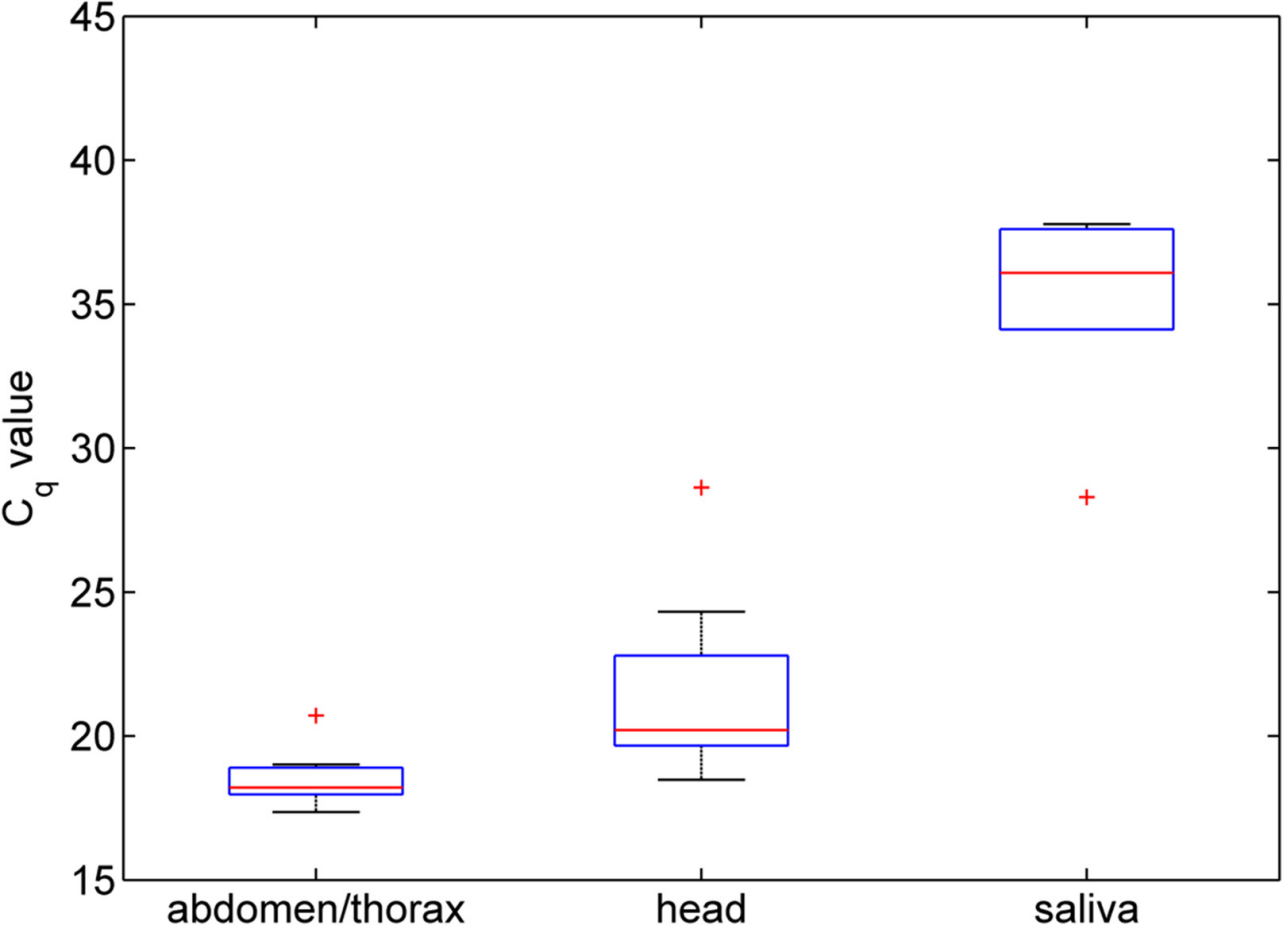
Observed C_q_ values for Schmallenberg virus in intrathoracically inoculated *Cx*. *pipiens* of the Brookwood line dissected at day 14 post-infection. The box-and-whisker plot shows the median (horizontal line), interquartile range (box), 1.5 times the interquartile range (whiskers) and any outliers (crosses).

All IT inoculated Brookwood line *Cx*. *pipiens* processed were found to contain SBV RNA. Individuals processed immediately following IT infection (n = 10) contained a median C_q_ of 21.6 (Range = 21.0–22.1), which was significantly less than those incubated for 14 days (n = 35) which contained a median C_q_ of 17.3 (Range = 16.4–30.1; Wilcoxon rank-sum test: *W* = 320, *P*<0.001) (Fig 1). In dissected, IT inoculated individuals processed at day 14 (n = 8) (Fig 2), the abdomen and thorax contained the greatest quantity of SBV RNA (median C_q_ = 18.2, Range = 17.4–20.7); followed by the head (median C_q_ = 20.2, Range = 18.5–28.6). Duplicated detection of SBV RNA was recorded in saliva of six Brookwood line mosquitoes processed, while two gave positive but unduplicated detection (median C_q_ = 36.1, Range = 28.3–37.8). Differences in SBV RNA load between body parts and saliva were statistically significant (χ^2^ = 15.86, *P*<0.001) and pairwise comparison identified significantly less SBV RNA in saliva than in the abdomen/thorax and head (χ^2^ = 18.14; P<0.001).

### Membrane-based Infections

All eight orally fed *Cx*. *pipiens* (five from the Brookwood line and three from the Caldbeck line) processed immediately following oral feeding on SBV/sheep blood suspension tested positive for SBV RNA (median C_q_ = 21.6, Range = 20.0–24.5). SBV RNA was detected in 27 of 322 *Cx*. *pipiens* from all lines incubated for 14 days following oral feeding (median C_q_ = 33.6, Range = 22.6–40.0). Using Fishers exact tests there no significant difference was found between the proportions of each line containing detectable SBV RNA. Median C_q_ values from individuals containing detectable SBV RNA were significantly different between the IT inoculated Brookwood line incubated for 14 days and the membrane fed Brookwood (W = 6; P<0.001), Caldbeck (W = 6; P<0.001) and Netherlands (W = 1; P<0.001) lines incubated for the same period following exposure. The maximum quantity of SBV RNA in any membrane-fed individual (C_q_ = 22.6) remained below the median quantity developed by *Cx*. *pipiens* during IT inoculation (C_q_ = 17.3) and peak RNA quantities in the Brookwood, Caldbeck and Netherlands lines fell outside the 95^th^ percentile of IT inoculated values for the Brookwood line. The highest SBV RNA C_q_ value within IT inoculated individuals was 22.6, which was identical to the lowest value returned by any membrane-fed individual (Table 3). Dissection of 18 *Cx*. *pipiens* of the Caldbeck line produced no evidence of replicating SBV in the abdomen/thorax and head with all C_q_ values recorded being >34 (Table H in S1 File).

**Table 3:** SBV RNA quantity detected in *Culex pipiens* from three colony lines fed a mixture of blood and tissue culture passaged virus or intrathoracically (IT) inoculated and then incubated for 14 days prior to processing.

C_q_	*Culex pipiens* colony line / Treatment			
	Brookwood (Membrane fed)	Caldbeck (Membrane fed)	Wageningen (Membrane fed)	Brookwood (IT inoculated)
**≥40**	92	71	113	0
**40–35**	9	3	6	0
**35–30**	7	6	3	1
**30–25**	4	3	1	0
**25–20**	2	2	0	5
**20–15**	0	0	0	29
**Total**	114	85	123	35
**Median C**_**q**_	33.3	32.6	35.4	17.3
**Range C**_**q**_	39–23.3	37.7–22.6	40–28.8	30–15.7
**RNA detection rate (%)**	19.3	16.5	8.1	100

## Discussion

This study demonstrated that three separate colony lines of *Cx*. *pipiens* originating from northern Europe were refractory to dissemination of SBV to the salivary glands following oral feeding on blood-virus mixtures through an artificial membrane. While 8.1–19.3% of *Cx*. *pipiens* lines tested at 14 days post-feeding contained detectable quantities of SBV RNA, the C_q_ values recorded were significantly higher than those recorded for IT inoculated individuals. These IT inoculated mosquitoes from the Brookwood line demonstrated a significant increase in SBV RNA detected from day 0 to day 14 post-inoculation and at day 14 had SBV RNA in their saliva. The smaller quantity of SBV RNA developed in membrane fed individuals in comparison to IT inoculation can be explained by the presence of midgut infection and/or escape barriers to infection and/or more general immune responses within the haemoceol, as documented in over vector-arbovirus relationships [26]. These barriers are either bypassed or overwhelmed during IT inoculation with the exception of those associated with the salivary glands. In addition, at least some of the higher C_q_ values generated (>35) may have occurred through the presence of inactivated SBV from the original blood meal remaining within the mosquito, which has been discussed previously [4]. The low proportion of infections in *Cx*. *pipiens* lines tested adds weight to recent studies that failed to detect SBV in field populations of mosquitoes [5,27,28], although other aspects of vectorial capacity known to vary among lines of this species (e.g. host preference and autogeny) may have a significant impact in the field. *Culicoides* therefore remain the only confirmed vector group for SBV to date [3,4,28,29].

Additional studies to confirm refractory status in *Cx*. *pipiens* populations could include attempts to isolate SBV from saliva of infected individuals. While SBV RNA was detected in IT inoculated individuals during the current study, this was relatively difficult to interpret in some cases due to the values being close to the cut-off value chosen for the assay. Isolation of virus would therefore provide additional security that what is being detected is live virus rather than trace inactivated virus from the original blood-virus meal. In addition, feeding of field mosquito populations on viraemic hosts would also provide a more natural system. One challenge in accomplishing this aim is the relatively short period of viraemia in ruminant hosts [30]. When paired with variation in field availability of mosquitoes and their blood-feeding responses, this makes studies extremely challenging to perform under biosecure conditions. In addition, a wide variety of virus, environmental and co-infection factors could all significantly influence transmission in the field as has been reviewed for both arboviruses and other pathogens [26,31]. This includes the potential for largely refractory populations to become co-infected with microfilariae which have the potential to disrupt the midgut integrity and lead to a far higher rate of competence for arboviruses [32,33].

This study also highlights the ongoing issues surrounding the classification of *Cx*. *pipiens*. Individuals of the pipiens, molestus and hybrid forms of *Cx*. *pipiens* have recently been recorded sharing the same habitat within Europe [13,15]. However, the fact that this genetic diversity was not substantially reduced within the colony lines and that these forms could be consistently supported within the Brookwood and Wageningen laboratory lines after initial colonization was unexpected. Interestingly, *Cx*. *torrentium*, which was inadvertently included in the original material for colonization from the Brookwood line, did not establish successfully and was not detected in subsequent screening, which may be due to a lack of stenogamy in this population. The different forms of *Cx*. *pipiens* have previously been characterized by ecological phenotypic characteristics such as autogeny, host preference and stenogamy that would be expected to provide selective advantages or disadvantages under colony conditions [34]. Further study is therefore required to both elucidate the taxonomic relationship and specific status of these two *Cx*. *pipiens* forms and define their status within the Brookwood and Wageningen colony lines. These investigations could include the characterization of phenotypic variation in relation to genotype.

While a recent study has highlighted an issue of cross-reaction when *Cx*. *torrentium* is processed alongside *Cx*. *pipiens* individuals using the CQ11 assay [15], we avoided this issue in the case of the Brookwood and Caldbeck lines by using a multi-stage screening of identity, with separation at species-level prior to definition of *Cx*. *pipiens* form identity. The Wageningen line used the alternative route of morphological identification to species level and then application of the CQ11 assay. A major consideration is whether vector competence studies are better served by isotype colonies that are often created to ensure phenotypic identity, as opposed to more natural lines created from taxonomically diverse field material, as used in the current study. The influence of hybridization on vector competence is challenging to assess, not least because the vector competence assays in the current study were not linked directly to species diagnostics for logistical reasons. Hence, while the Caldbeck line (which contained only *Cx*. *pipiens* f. pipiens, even in the original field material) is highly likely to be representative of this form, results from the Brookwood line are more difficult to interpret. The fact that these lines and the additional Wageningen line originating from The Netherlands failed to support full SBV dissemination, however, would suggest that the presence of hybrids in tested populations does not substantially influence vector competence for SBV, in contrast to WNV [17]. Further studies to examine this issue directly with zoonotic pathogens would be useful in elucidating the potential role that these hybrids might play in arbovirus outbreaks in northern Europe.

Until relatively recently, testing the vector competence of UK mosquitoes for arboviruses has been a relatively low priority. Recent emergence of *Culicoides*-borne pathogens [6,35], incursions of exotic mosquitoes in northern Europe [36] and outbreaks of arboviruses in the southern Mediterranean [37], have heightened awareness of this area and led to dedicated risk assessments of mosquito-borne arboviruses for the UK [9,38] and most recently the demonstration that *Ochlerotatus detritus* (Haliday) can support full replication of Japanese encephalitis virus following membrane-based feeding [39]. This highlights that testing a range of potential vector species is of significant importance in risk assessment studies for arbovirus transmission. The establishment of UK-derived colony lines of *Cx*. *pipiens* within this study contributes to providing fundamental data regarding not only vector competence, but a wide-array of other phenotypic variation that may influence vectorial capacity. Colonies allow the standardization of populations tested according to age, rearing conditions and a wide-variety of other factors that can otherwise influence studies [40]. The Brookwood and Caldbeck colony lines can be provided to global workers for such studies under the Biotechnology and Biological Sciences Research Council National Capability initiative, through contact with the corresponding author.

## Supporting Information

S1 File Supporting datasets for studies. Duplicated C_q_ values from *Cx*. *pipiens* mosquitoes intrathoracically (Brookwood line) inoculated with Schmallenberg virus and then processed immediately using sqPCR (**Table A in S1 File**). Duplicated C_q_ values from *Cx*. *pipiens* mosquitoes (Brookwood line) intrathoracically inoculated with Schmallenberg virus and then processed following a 14 day incubation period using sqPCR (**Table B in S1 File**). Duplicated C_q_ values from *Cx*. *pipiens* mosquitoes (Brookwood line) intrathoracically inoculated with Schmallenberg virus and then processed following a 14 day incubation period. Saliva from each mosquito was collected using a glass capillary tube and insecticidal treatment and the body was then dissected into head and abdomen/thorax before processing using sqPCR (**Table C in S1 File**). C_q_ values from *Cx*. *pipiens* mosquitoes (Brookwood line) fed through a membrane on a Schmallenberg virus/blood suspension and then processed immediately using sqPCR (**Table D in S1 File**). C_q_ values from *Cx*. *pipiens* mosquitoes (Brookwood line) fed through a membrane on a Schmallenberg virus/blood suspension and then processed after a 14 day incubation period using sqPCR. A total of 92 samples returned no C_q_ value or a value >40 (**Table E in S1 File**). C_q_ values from *Cx*. *pipiens* mosquitoes (Caldbeck line) fed through a membrane on a Schmallenberg virus/blood suspension and then processed immediately using sqPCR (**Table F in S1 File**). C_q_ values from *Cx*. *pipiens* mosquitoes (Caldbeck line) fed through a membrane on a Schmallenberg virus/blood suspension and then processed after a 14 day incubation period using sqPCR. A total of 71 samples returned no C_q_ value or a value >40 (**Table G in S1 File**). C_q_ values from *Cx*. *pipiens* mosquitoes (Caldbeck line) fed through a membrane on a Schmallenberg virus/blood suspension and then processed following a 14 day incubation period. Saliva from each mosquito was collected using a glass capillary tube and insecticidal treatment and the body was then dissected into head and abdomen/thorax before processing using sqPCR (**Table H in S1 File**). C_q_ values from *Cx*. *pipiens* mosquitoes (Wageningen line) fed through a membrane on a Schmallenberg virus/blood suspension and then processed after a 14 day incubation period using sqPCR. A total of 113 samples returned no C_q_ value or a value >40 (**Table I in S1 File**).
(DOCX)
