# Supplementary material for: Testing of UK Populations of Culex pipiens L. for Schmallenberg Virus Vector Competence and Their Colonization
Source: PLoS One. 2015 Aug 20;10(8):e0134453. doi: 10.1371/journal.pone.0134453 (PMC4546389; doi:10.1371/journal.pone.0134453)
Supplement: S1 File — Duplicated Cq values from Cx. pipiens mosquitoes intrathoracically (Brookwood line) inoculated with Schmallenberg virus and then processed immediately using sqPCR (Table A in S1 File). Duplicated Cq values from Cx. pipiens mosquitoes (Brookwood line) intrathoracically inoculated with Schmallenberg virus and then processed following a 14 day incubation period using sqPCR (Table B in S1 File). Duplicated Cq values from Cx. pipiens mosquitoes (Brookwood line) intrathoracically inoculated with Schmallenberg virus and then processed following a 14 day incubation period. Saliva from each mosquito was collected using a glass capillary tube and insecticidal treatment and the body was then dissected into head and abdomen/thorax before processing using sqPCR (Table C in S1 File). Cq values from Cx. pipiens mosquitoes (Brookwood line) fed through a membrane on a Schmallenberg virus/blood suspension and then processed immediately using sqPCR (Table D in S1 File). Cq values from Cx. pipiens mosquitoes (Brookwood line) fed through a membrane on a Schmallenberg virus/blood suspension and then processed after a 14 day incubation period using sqPCR. A total of 92 samples returned no Cq value or a value >40 (Table E in S1 File). Cq values from Cx. pipiens mosquitoes (Caldbeck line) fed through a membrane on a Schmallenberg virus/blood suspension and then processed immediately using sqPCR (Table F in S1 File). Cq values from Cx. pipiens mosquitoes (Caldbeck line) fed through a membrane on a Schmallenberg virus/blood suspension and then processed after a 14 day incubation period using sqPCR. A total of 71 samples returned no Cq value or a value >40 (Table G in S1 File). Cq values from Cx. pipiens mosquitoes (Caldbeck line) fed through a membrane on a Schmallenberg virus/blood suspension and then processed following a 14 day incubation period. Saliva from each mosquito was collected using a glass capillary tube and insecticidal treatment and the body was then dissected into head and abd [file pone.0134453.s001.docx]

**Table S1a.** Duplicated C_q_ values from *Cx. pipiens* mosquitoes intrathoracically inoculated with Schmallenberg virus and then processed immediately using sqPCR.

| **C_q_1** | **C_q_2** | **C_q_1** | **C_q_2** |
| --- | --- | --- | --- |
| 21.0 | 21.0 | 21.5 | 21.5 |
| 21.3 | 21.6 | 21.8 | 21.7 |
| 21.6 | 21.5 | 22.1 | 21.9 |
| 22.1 | 22.2 | 22.1 | 22.3 |
| 21.3 | 21.6 | 21.6 | 21.6 |

**Table S1b.** Duplicated C_q_ values from *Cx. pipiens* mosquitoes intrathoracically inoculated with Schmallenberg virus and then processed following a 14 day incubation period using sqPCR.

| **C_q_1** | **C_q_2** | **C_q_1** | **C_q_2** | **C_q_1** | **C_q_2** | **C_q_1** | **C_q_2** |
| --- | --- | --- | --- | --- | --- | --- | --- |
| 22.6 | 22.9 | 17.1 | 17.6 | 16.8 | 17.0 | 17.3 | 17.8 |
| 16.9 | 17.1 | 16.9 | 17.0 | 20.5 | 20.7 | 15.9 | 16.5 |
| 17.1 | 17.5 | 16.9 | 17.0 | 19.2 | 19.3 | 16.1 | 16.1 |
| 16.3 | 16.5 | 17.8 | 17.9 | 18.1 | 18.6 | 16.3 | 16.5 |
| 17.0 | 17.5 | 17.8 | 18.4 | 16.4 | 16.7 | 17.7 | 17.8 |
| 21.4 | 21.6 | 16.4 | 16.8 | 17.3 | 17.3 | 18.3 | 18.4 |
| 15.7 | 16.0 | 30.0 | 30.1 | 17.3 | 17.6 | 19.7 | 20.1 |
| 21.2 | 21.3 | 21.7 | 21.8 | 17.8 | 18.0 | 16.6 | 16.7 |
| 17.4 | 17.5 | 16.7 | 16.8 | 17.3 | 17.2 |  |  |

**Table S1c.** Duplicated C_q_ values from *Cx. pipiens* mosquitoes intrathoracically inoculated with Schmallenberg virus and then processed following a 14 day incubation period. Saliva from each mosquito was collected using a glass capillary tube and insecticidal treatment and the body was then dissected into head and abdomen/thorax before processing using sqPCR.

| **Saliva** | | **Head** | | **Abdomen and Thorax** | |
| --- | --- | --- | --- | --- | --- |
| **C_q_1** | **C_q_2** | **C_q_1** | **C_q_2** | **C_q_1** | **C_q_2** |
| 37.6 | No Ct | 24.0 | 24.6 | 18.2 | 18.5 |
| 34.6 | 35.1 | 20.1 | 20.3 | 17.9 | 18.2 |
| 37.8 | 36.8 | 19.2 | 19.9 | 17.9 | 17.9 |
| No Ct | No Ct | 18.4 | 18.6 | 17.3 | 17.4 |
| No Ct | 37.8 | 20.9 | 21.7 | 17.9 | 18.3 |
| No Ct | No Ct | 20.0 | 20.4 | 18.6 | 19.0 |
| 33.8 | 34.5 | 28.4 | 28.9 | 20.7 | 20.8 |
| 28.6 | 28.0 | 19.7 | 19.8 | 18.8 | 19.2 |

**Table S1d.** C_q_ values from *Cx. pipiens* mosquitoes (Brookwood line) fed through a membrane on a Schmallenberg virus/blood suspension and then processed immediately using sqPCR.

| **C_q_1** |
| --- |
| 20.7 |
| 21.0 |
| 20.7 |
| 22.3 |
| 20.0 |

**Table S1e.** C_q_ values from *Cx. pipiens* mosquitoes (Brookwood line) fed through a membrane on a Schmallenberg virus/blood suspension and then processed after a 14 day incubation period using sqPCR. A total of 92 samples returned no C_q_ value or a value >40.

| **C_q_1** | **C_q_1** | **C_q_1** | **C_q_1** | **C_q_1** |
| --- | --- | --- | --- | --- |
| 23.3 | 29.2 | 33.0 | 36.6 | 38.3 |
| 24.1 | 31.1 | 33.6 | 36.7 | 39.0 |
| 26.1 | 32.0 | 33.6 | 37.5 |  |
| 26.9 | 32.5 | 35.5 | 38.2 |  |
| 27.7 | 32.5 | 35.5 | 38.3 |  |

**Table S1f.** C_q_ values from *Cx. pipiens* mosquitoes (Caldbeck line) fed through a membrane on a Schmallenberg virus/blood suspension and then processed immediately using sqPCR.

| **C_q_1** |
| --- |
| 23.9 |
| 22.8 |
| 24.5 |

**Table S1g.** C_q_ values from *Cx. pipiens* mosquitoes (Caldbeck line) fed through a membrane on a Schmallenberg virus/blood suspension and then processed after a 14 day incubation period using sqPCR. A total of 71 samples returned no C_q_ value or a value >40.

| **C_q_1** | **C_q_1** | **C_q_1** |
| --- | --- | --- |
| 22.6 | 30.5 | 34.6 |
| 24.5 | 32.4 | 35.2 |
| 27.4 | 32.7 | 35.4 |
| 27.9 | 33.4 | 37.7 |
| 29.9 | 33.7 |  |

**Table S1h.** C_q_ values from *Cx. pipiens* mosquitoes (Caldbeck line) fed through a membrane on a Schmallenberg virus/blood suspension and then processed following a 14 day incubation period. Saliva from each mosquito was collected using a glass capillary tube and insecticidal treatment and the body was then dissected into head and abdomen/thorax before processing using sqPCR.

| **Saliva** | **Head** | **Abdomen and Thorax** |
| --- | --- | --- |
| **C_q_1** | **C_q_1** | **C_q_1** |
| No Ct | No Ct | No Ct |
| 37.8 | No Ct | 33.0 |
| 34.6 | 37.3 | No Ct |
| 35.3 | No Ct | No Ct |
| No Ct | No Ct | No Ct |
| No Ct | No Ct | No Ct |
| No Ct | No Ct | No Ct |
| No Ct | No Ct | No Ct |
| No Ct | No Ct | No Ct |
| No Ct | No Ct | No Ct |
| No Ct | No Ct | No Ct |
| No Ct | No Ct | No Ct |
| No Ct | No Ct | No Ct |
| No Ct | 34.4 | No Ct |
| No Ct | 36.3 | No Ct |
| No Ct | No Ct | No Ct |
| No Ct | No Ct | No Ct |
| No Ct | No Ct | No Ct |

**Table S1i.** C_q_ values from *Cx. pipiens* mosquitoes (Wageningen line) fed through a membrane on a Schmallenberg virus/blood suspension and then processed after a 14 day incubation period using sqPCR. A total of 113 samples returned no C_q_ value or a value >40.

| **C_q_1** | **C_q_1** |
| --- | --- |
| 28.8 | 35.6 |
| 33.3 | 36.0 |
| 33.6 | 37.8 |
| 34.8 | 39.8 |
| 35.3 | 40.0 |
